# How Sex Shapes Facial Morphology in Adults: A 3D Geometric Morphometric Study

**DOI:** 10.3390/diagnostics16050712

**Published:** 2026-02-27

**Authors:** Riccardo Solazzo, Daniele Maria Gibelli, Alice Alderighi, Claudia Dolci, Chiarella Sforza, Annalisa Cappella

**Affiliations:** 1 Laboratory of Functional Anatomy of the Stomatognathic System (LAFAS), Department of Biomedical Sciences for Health, University of Milan, 20133 Milan, Italy; riccardo.solazzo@unimi.it (R.S.); daniele.gibelli@unimi.it (D.M.G.); alicealderighi97@gmail.com (A.A.); chiarella.sforza@unimi.it (C.S.); 2 U.O. Laboratory of Applied Morphology, IRCCS Policlinico San Donato, 20097 San Donato Milanese, Italy; annalisa.cappella@unimi.it; 3 Department of Biomedical Sciences for Health, University of Milan, 20133 Milan, Italy

**Keywords:** sexual dimorphism, anatomical variation, facial morphology, anthropometry, 3D imaging, geometric morphometrics

## Abstract

**Background/Objectives**: An accurate description of facial sexual dimorphism is essential in clinical, forensic, and anthropological contexts to support accurate diagnosis of craniofacial dysmorphisms and differences, treatment planning and evaluation, as well as biological profiling, craniofacial reconstruction, and personal identification. This study investigates sexual dimorphism of the facial soft tissues in a sample of healthy Italian adults, providing reference data and deepening our understanding of normal craniofacial variation. **Methods**: Three-dimensional stereophotogrammetric facial images of 342 Italian adults (172 males and 170 females; 18–40 years old) were analyzed using a 3D spatially dense geometric morphometric approach to assess both shape and form. Principal Component Analysis (PCA) and Partial Least Squares Regression (PLSR) were used to explore facial variation and to quantify sex-related differences. **Results**: Centroid size was significantly larger in males. While PCA revealed that sex is a significant factor in facial shape and form variation, PLSR highlighted the existence of significant associations between sex and both shape and form. Color-coded morphometric maps underlined the most sexually dimorphic traits: males exhibited bigger faces with deep-set eyes and central facial projection extending from the supraorbital rims to the chin, whereas females display smaller faces with fuller cheeks, and a more vertical forehead profile. **Conclusions**: While our results are consistent with those of previous studies, our study revealed important, distinctive group-specific traits: flatter labiomandibular folds in males and wider temples and fuller cheeks in the infraorbital region extending to zygomatic and mandibular areas in females. Thus, this study provides high-resolution reference data supporting related applications.

## 1. Introduction

The human face conveys a lot of the biological and social information of a person, including age, sex, biogeographical origin, and health [1]. Facial morphology is the result of a complex interplay between the genome and multiple endogenous (e.g., hormonal) and exogenous (e.g., environmental and social) factors [2]. From this standpoint, biological sex is considered a major contributing factor to facial morphology, and the face is one of the most sexually dimorphic anatomical regions in humans [3]. However, the manifestation of sexual dimorphism is population-specific, as comparative studies between European, African, and Asian populations have indeed highlighted both shared and divergent sexually dimorphic features [3,4]. Therefore, a population-specific detailed characterization of facial sexual dimorphism, including information about its nature, extent, onset, variability, and expression, is relevant to various disciplines, such as clinical anatomy and dysmorphology [5], reconstructive and maxillofacial surgery [6], and anthropology and forensic sciences [7]. While these data assist in clinical surgical planning (e.g., gender-affirming surgeries) by defining realistic, sex-consistent outcomes [8], they are also useful for distinguishing between ‘normal’ and ‘dysmorphic’ craniofacial variations. In forensics, such models enhance the accuracy of biological profiling and craniofacial reconstruction [9], supporting the personal identification of unknown deceased persons or living subjects captured by video surveillance systems, such as closed-circuit television (CCTV) cameras. Moreover, in anthropology, characterizing sex-related facial variation contributes to comparative and evolutionary studies on craniofacial diversity and population differentiation [3,4]. Finally, integrating these morphometric parameters into digital technologies—such as biometric facial identification systems, AI-driven facial modeling, and virtual human simulations—could further extend the applicability of these findings to emerging interdisciplinary fields, including digital forensics [10].

The consensus on facial sexual dimorphism is that most sexually dimorphic traits become more pronounced during puberty under the influence of sex hormones [3], but previous research has reported sex differences prior to puberty [11,12]. Further, since facial sexual dimorphism is a dynamic rather than static phenomenon, its manifestation continues to evolve throughout life under the influence of age-related processes such as skeletal remodeling, soft tissue volume loss, and changes in facial fat distribution [13].

Historically, facial sexual dimorphism has been evaluated by means of sparse landmark-based approaches, including direct and digital anthropometry [11]. While these methods are still considered the gold standard, they offer limited information on the three-dimensional (3D) morphology and complexity of the face, as they typically rely on a set of two-dimensional parameters (e.g., linear measurements, angles, and ratios) [11]. In addition, these methods fail to provide an accurate representation of those areas where landmarks are few and poorly informative (e.g., cheeks and forehead) and they require prior knowledge or expectation of which features possess the greatest sex differences [14]. Similarly, geometric morphometric (GMM) approaches based on sparse landmarks, despite retaining the 3D characteristics of the analyzed structure, provide limited information in landmark-deficient region [15]. The increased utilization of 3D imaging technologies for facial soft tissues (e.g., stereophotogrammetry, laser scanning, and structured-light scanning) allows the acquisition of high-resolution facial surfaces suitable for detailed morphometric analysis [16,17]. The advent of spatially dense GMM approaches applied to 3D images has transformed the study of craniofacial morphology by enabling the analysis of thousands of homologous points across the entire surface of interest, allowing the comprehensive assessment of subtle morphological variations, including age and sex-related differences. Despite these advances, relatively few studies have applied spatially dense GMM approaches to simultaneously examine shape and form sex-related differences in Caucasoid populations [6,18]. The analysis of both shape and form provides complementary insights: form incorporates overall size, a strong contributor to male–female facial differences [6,18], while shape isolates pure morphological differences independent of size [19].

To the best of our knowledge, no previous study has applied a spatially dense morphometric approach to a group of Italian adults, as earlier investigations have primarily relied on two-dimensional imaging techniques [20] or sparse 3D landmark-based approaches [8,21]. Extending our previous work on Italian juveniles ranging from 3 to 18 years old [15], this study investigates facial sexual dimorphism in adults, advancing the morphological description of such phenomenon in our reference population and providing mapping regions of pronounced dimorphism and high-resolution group-specific reference data valuable for forensic and anthropological applications.

## 2. Materials and Methods

### 2.1. Study Sample

The study sample comprised 342 stereophotogrammetric 3D facial images of Italian adults. Specifically, the sample included White subjects from northwest regions of Italy aged between 18 and 40 years: 172 males (23.2 ± 5.8 years) and 170 females (25.7 ± 7.2 years). Because males and females showed slightly different mean ages and standard deviations, between-group differences in age were tested using a Mann–Whitney U test after checking data normality and homoscedasticity.

The 3D images were acquired with two validated instruments [22] (Vectra M3 and Vectra H2; Canfield Scientific Inc., Parsippany, NJ, USA). The exclusion criteria were poor quality of the 3D image, incomplete depiction of the face, non-neutral facial expression of the subject, presence of facial hair (beard and mustaches), and presence of pathologies, traumas or previous surgeries affecting the craniofacial district.

The present study is part of a wider project approved by the local ethics committee of the University of Milan (protocol 19/24; 13 February 2024), and it was conducted in accordance with the Declaration of Helsinki [23]. Written informed consent was obtained from all the participants.

### 2.2. Geometric Morphometric Analysis

Facial sexual dimorphism was investigated using a spatially dense geometric morphometric approach. According to previous background research [19], each 3D facial image was processed using the MeshMonk pipeline (latest stable release available as November 2022) [19]: an anthropometric mask of 7160 vertices was non-rigidly registered onto all facial surfaces using an iterative closest point algorithm, initially guided by manually annotated landmarks whose reliability was previously validated [15]. Analogously, the reliability of the non-rigid mapping procedure has already been verified [24]. The non-rigid alignment ensured that all faces were represented by the same set of homologous quasi-landmarks [25]. To evaluate the impact of sex on facial morphology, subsequent analyses were restricted to the symmetric component of facial shape and form. This component is generally considered to reflect structured differences between males and females arising from genetic and developmental factors underlying sexual dimorphism. In contrast, the asymmetric component, not analyzed in the present study, captures random, individual-specific variations attributed to ‘noise’ in the development of the subject [26]. Symmetrization was accomplished by reflecting each dense quasi-landmark’s configuration along the x axis, changing the sign of the x coordinates and relabeling the paired landmarks as the corresponding one on the opposite side. The reflected configuration was superimposed on the original one through least-squares Procrustes superimposition and the average of each of the quasi-landmarks was computed to obtain the symmetrical version of the face.

To analyze sex-related differences in both shape and form, two Generalized Procrustes Analyses (GPAs) were performed. The GPA for the shape analysis removed differences due to translation, rotation, and scale by aligning all faces to a common shape configuration and scaling them to the average size of the sample [27]. This allowed regression coefficients from subsequent analyses to be expressed in millimeters, enabling a direct quantification of shape differences. The GPA for the form removed only translation and rotation, preserving the original size of each face [27]. The centroid size (CS), a measure of overall facial dimensions, was calculated for each face as the square root of the summed squared distances of all quasi-landmarks from their centroid, and it was used as a proxy for facial size [27]. Differences between males and females CS were tested using Student’s *t*-test, following verification of normality and homogeneity of variance through Kolmogorov–Smirnov and Levene’s tests, respectively. The percentage difference in facial size (CS) between the two sexes was calculated according to Cole and Altman [28]. Subsequently, Principal Component Analysis (PCA) and Partial Least Squares Regression (PLSR) were applied to both shape and form datasets. Specifically, PCA was used to reduce data dimensionality and explore the major axes of variation in facial shape and form, and Principal Components (PCs) accounting for at least 1% of variance were retained. PLSR evaluated the effect of sex on facial shape and form morphology, and sex was coded as a binary predictor (female = 0; male = 1). Despite the restricted age range of this study cohort, age was included as a covariate in both shape and form PLSR analyses to account for potential age-related facial changes persisting into early adulthood. In the shape PLSR analysis, centroid size was incorporated as an additional covariate to eliminate residual allometric effects, thereby ensuring that the regression coefficients isolated pure shape variations independent of scale. Conversely, centroid size was excluded from the form analysis, as form inherently integrates size-related information into the morphological assessment. To quantify the proportion of total facial variance ascribable to sex and the specific covariates (age and size for shape PLSR; age for form PLSR), the R^2^ coefficient [29], a multivariate effect size statistic, was calculated. The statistical significance of these associations was subsequently assessed through 1000 permutations.

The results of the PCA were illustrated by producing a scatterplot of the first two PCs, which captured the highest percentage of morphological variance. Related facial morphs were generated to visualize these effects through heatmaps. Furthermore, PLSR results were visualized using vertex-wise heatmaps of the regression coefficients mapped onto the average facial surface. These maps were color-coded to represent the direction and magnitude of the observable morphological differences between the average male and female faces. All statistical analyses and visualizations were conducted using MATLAB R2024a (The Mathworks Inc., Natick, MA, USA).

## 3. Results

The Mann–Whitney U test revealed no statistically significant difference in the age distribution between the male and female subgroups (U = 16,175; *p* = 0.086). Consequently, age is not expected to bias the findings regarding sexual dimorphism in facial shape and form.

### 3.1. Sex Dimorphism of Size

**Figure 1:**
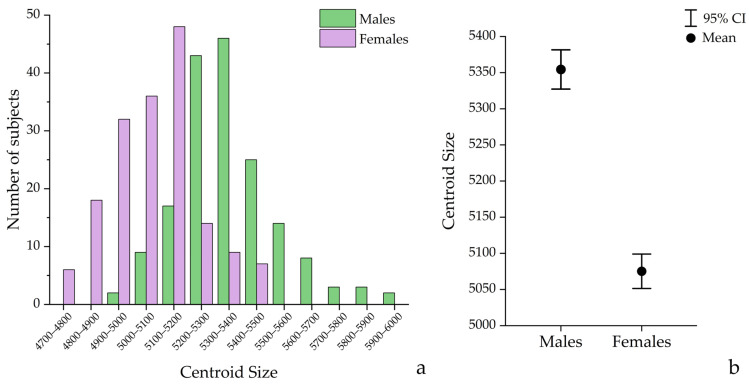
Sex difference in facial centroid size. (**a**) shows the distribution of centroid sizes; (**b**) displays the means (circle) ± 95% Confidence Interval (CI; error bars).

The centroid size, a measure of overall facial size, showed that the data were normally distributed and homoscedastic for both males and females. Student’s *t*-test revealed that males (5351.87 ± 180.15) possessed a significantly greater facial size than females (5075.16 ± 156.76) (t_(340)_ = 15.24, *p* < 0.001), with a 5.3% difference in mean facial size between the two sexes. Detailed distributions and average centroid sizes for both groups are presented in Figure 1.

### 3.2. Sex Dimorphism of Shape

**Figure 2:**
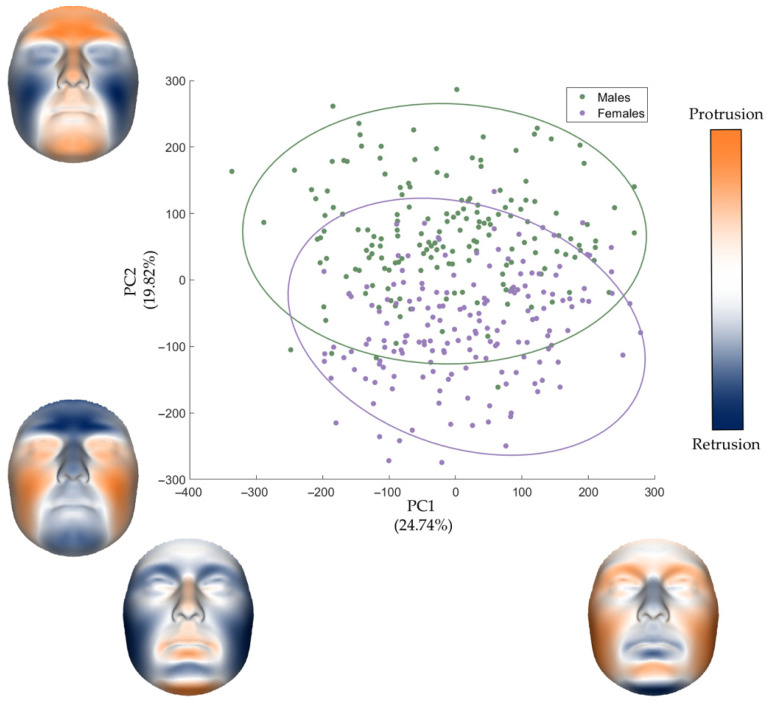
Scatterplot of PC1 and PC2 with related morphs of the effects of each PC on facial shape morphology. The ellipses represent the 95% data ellipses for each sex. In the color-coded maps, blue represents retrusion while orange represents protrusion.

The PCA for shape analysis revealed that the first 15 PCs accounted for almost 85% of the total morphological variance, with PC1 and PC2 alone explaining approximately 45% (Table 1). As shown in Figure 2, PC1 captured variations in overall facial dimensions without clear separation between the sexes. Despite the removal of size in the GPA, this pattern likely reflect the residual allometric shape variation [27]. Specifically, positive PC1 scores were associated with shorter, wider faces characterized by less projecting perinasal and perioral regions, alongside increased protrusion of the orbital, malar and chin regions, whereas negative PC1 scores exhibited the opposite morphological manifestations. In contrast, PC2 partially distinguished the two sexes by capturing morphological differences in regions typically associated with sexual dimorphism. Higher, positive PC2 scores corresponded to masculine features, such as squarer facial outline, prominent brow ridges, and an anteriorly projecting midface and chin. Lower, negative PC2 scores were instead associated with feminine traits, including rounder facial contours, fuller cheeks, and a flatter profile.

**Figure 3:**
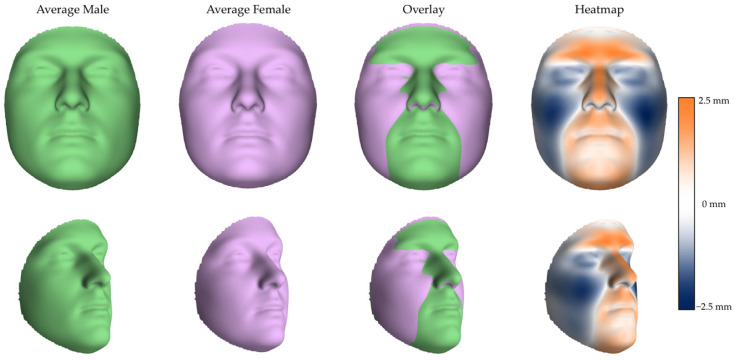
Average shapes of male (green) and female (purple) faces with the related overlay and heatmap in frontal (**upper row**) and lateral (**lower row**) views. In the color-coded maps (heatmap), orange areas are those where the average male face protrudes compared to the average female face, while blue areas represent the opposite trend.

The PLSR analysis confirmed a significant effect of sex on facial shape (R^2^ = 0.1035, *p* < 0.001), after accounting for participants’ age and centroid size (R^2^ = 0.0154; R^2^ = 0.0261, respectively). Approximately 10.4% of the total facial shape variance was independently attributable to sex. The average male and female facial configurations, their superimposition, and the corresponding color-coded map of sexual dimorphism are illustrated in Figure 3. The male facial phenotype exhibited a greater projection of the lower forehead and brow ridges, a more prominent nasal dorsum, and an increased protrusion of the cutaneous upper lip and lower vermilion. The chin was notably broader and more elongated, in combination with more laterally displaced nasal alae. The female facial phenotype exhibited a more vertical forehead profile and more projecting orbital regions. Further distinctive traits included fuller infraorbital cheeks, with a gradual fading of projection toward the zygomatic and buccal areas, and a mild protrusion of the temporal region.

### 3.3. Sex Dimorphism of Form

**Figure 4:**
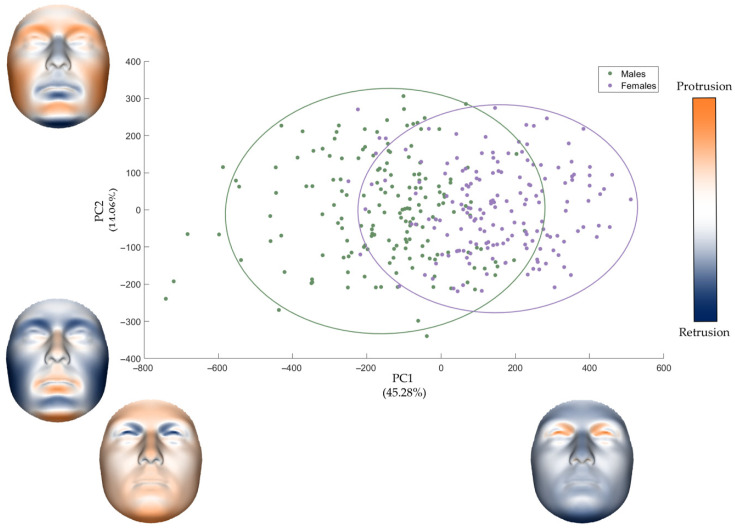
Scatterplot of PC1 and PC2 with related morphs of the effects of each PC on facial form morphology. The ellipses represent the 95% data ellipses for each sex. In the color-coded maps, blue represents retrusion while orange represents protrusion.

Concerning the analysis of facial form, the first nine PCs explained more than 85% of the total variance, with PC1 and PC2 accounting for nearly 60% (Table 2). As illustrated in Figure 4, the male and female samples showed partial separation along PC1, which primarily captured differences in overall facial dimensions. Specifically, positive PC1 scores were associated with more feminine faces, characterized by smaller and shorter dimensions. A distinctive feature at positive scores was the protrusion of the eyes and the medial portion of the upper eyelid. Negative PC1 scores corresponded to the more robust masculine phenotype, with the opposite morphological trends observed towards negative values. The morphological facial features captured by PC2 mainly influenced the length and width of the face, along with variations in the projection of the perinasal and perioral regions. In detail, positive PC2 scores corresponded to larger but shorter faces, featuring posteriorly positioned perioral and perinasal regions. Negative PC2 scores exhibited the opposite facial phenotype.

**Figure 5:**
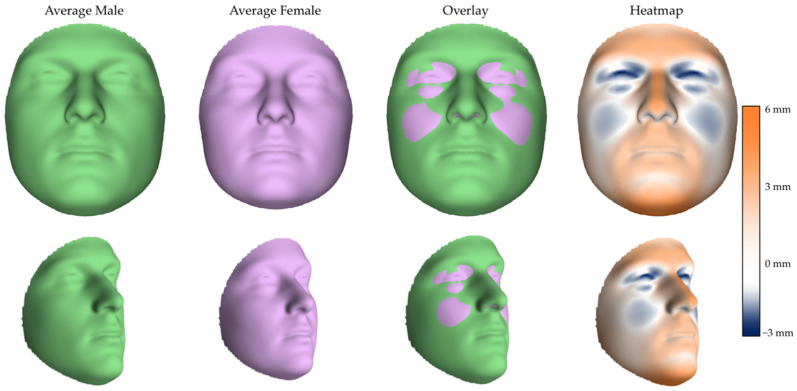
Average forms of male (green) and female (purple) faces with the related overlay and heatmap in frontal (**upper row**) and lateral (**lower row**) views. In the color-coded maps (heatmap), orange areas are those where the average male face protrudes compared to the average female face, while blue areas represent the opposite trend.

The PLSR analysis revealed that sex had a highly significant effect on facial form (R^2^ = 0.2416, *p* < 0.001), after adjusting for age (R^2^ = 0.0154) but not size. In this model, sex accounted for approximately 24.2% of total facial form variance, more than twice the explanatory power observed in the shape-only analysis. The morphological differences, shown in Figure 5, highlight the combined impact of size and shape: male phenotypes exhibited longer and wider faces, with greater projection of the lower forehead, nasal dorsum, and perioral region, except for the upper chin, which showed less projection. Female phenotypes, in contrast, were characterized by outwardly displaced orbital regions and increased projection of the infraorbital cheeks.

## 4. Discussion

The present study investigated facial sexual dimorphism in a cohort of Italian adults using a spatially dense GMM approach applied to both facial shape and form. To our knowledge, this represents the first investigation specifically designed to compare dimorphism in both these morphological aspects within this particular population. Direct comparisons with previous investigations in Italians are currently limited by significant methodological discrepancies, such as the use of sparse landmarks or traditional anthropometry [8,20,21]. Despite differences in demographic characteristics [4,12,25,30,31,32,33], methodological approaches [12], image processing [34], and quantification of sex dimorphism [14], our findings remain fundamentally consistent with the broader literature reported for other Caucasoid and European-descendent populations (see for example, [6,11,14,25,33]).

Our results indicate that male facial soft tissues are 5.3% greater in size than female ones, in line with Ferrario et al. [21] and Bannister et al. [6], who reported facial size differences ranging from 6% to 7.3%. This size-related divergence is a well-established dimorphic trait likely driven by multiple factors [18]. Facial shape and size correlate allometrically with features such as overall body dimensions, particularly height, which is typically greater in males [35]. Indeed, the greater average height in males is often reflected in a larger, more robust skull (skeletal robusticity), which directly contributes to increased facial size [36,37]. Other evolutionary and physiological factors may further explain the size discrepancies observed between the faces of males and females: differences in masticatory and respiratory systems [4,38] alongside the influence of sex hormones [39,40]. The impact of size on phenotypic differentiation is a crucial insight from our study: by incorporating size into the morphological assessment (form analysis), sex-related differences were substantially amplified. While sex accounted for 10.4% of shape variance, this more than doubled to 24.2% in the form model, demonstrating that both global size and localized morphological features are not merely independent variables but are fundamental, integrated components of human facial sexual dimorphism.

The application of the 3D spatially dense GMM approach enabled for a high-resolution characterization of the facial phenotypes associated with each sex. Our results confirm that sex-related differences are observable across nearly the entire face. Notable exceptions where significant differences did not emerge were localized to small areas like the columella, upper vermilion, and the mentolabial sulcus, consistent with findings reported for Italian or other Caucasoid/European-descendant populations (Table 3). Comprehensive details regarding additional relevant studies, including those employing methodologies distinct from 3D spatially dense GMMs, are provided in Supplementary Table S1. Table S1 also includes specific information on sample size, sex distribution, and age ranges across the cited literature.

The majority of existing studies have characterized facial sexual dimorphism through the analysis of shape rather than form, and only a limited number of investigations have quantified the actual contribution of sex to facial variance (summarized in Table 3). In accordance with the findings of Bannister et al. [6], we identified a substantial contribution of sex to facial form (30% and 24% in their study and ours, respectively), further supporting the hypothesis that size-related morphological aspects consistently amplify sexual divergence. Concerning facial shape, Claes et al. [25], using Bootstrapped Response-based Imputation Modeling (BRIM), found that sex explained almost 13% of total morphological variations in an ethnically admixed sample (Brazilian, Europeans, and West African). In our cohort, the sex effect evaluated through PLSR was slightly lower (approximately 10%) but remained comparable to the results of Claes et al. [25] and our previous investigation onto older adolescents (12%) [15]. In contrast, Bannister et al. [6] reported a lower contribution (6%) of sex to facial shape by using PLSR analysis, and Da Silva et al. [14] found an even smaller sex effect through a ‘sex-on-shape regression’ analysis: 3.5% and 1.7% for the allometric and non-allometric shape variations. These discrepancies in the reported magnitude of sexual dimorphism can likely arise from two main factors: sample characteristics and statistical methodologies. Regarding population effects, variations are evident when comparing generically defined ‘European-descendant’ cohorts with specific ethnic groups. For instance, it is well-established that South American populations tend to exhibit higher levels of facial sexual dimorphism compared to European ones, which may explain the higher variance explained in the study by Claes et al. [25]. Thus, we can hypothesize that differences in sample characteristics, as well as in the statistical analyses used to quantify the effect of sex, may explain the differences in the results. Moreover, the R^2^ values describing the contribution of sex to facial form and shape indicate that a substantial proportion of variance remains unexplained. This is likely attributable to covariates other than age and size, including variables not considered in the present study, such as body composition and BMI, hormonal profile, or specific genetic factors. In our sample, size accounted for a minimal proportion of facial shape variance (2.6%), while age explained an even lower percentage (1.5%) across both facial shape and form. The negligible and non-significant contribution of age is likely a consequence of the narrow age range investigated. While this focus reduces the potential confounding effect of age-related morphological changes, it inherently limits the generalizability of our findings to other age cohorts. Indeed, while sexually dimorphic traits are often emphasized during growth in subadults [15,42], the literature suggests an overall decline in the strength of dimorphism in older adults [33,43]. This reduction may be driven by many factors, such as bone resorption, or the effects of gravity, decreased tissue elasticity, and the redistribution of subcutaneous fat [13]. However, due to the demographic constraints of our sample, we were unable to explicitly verify this phenomenon.

The morphological description of the sex-related differences in Italians pointed out a peculiar combination and expression of traits that altogether define a group-specific pattern. Indeed, studies applying a GMM approach outperform those based on sparse landmarks by providing more detailed anatomical data. Across the literature, a consensus has emerged (Table 3): males generally possess larger faces characterized by supraorbital bossing, posteriorly sloped foreheads, protruding noses and upper lips, and robust chins, whereas females exhibit smaller, rounder faces with flatter and vertical profiles (forehead), fuller cheeks in the infraorbital region, and tapered chins. Consequently, we will discuss sexually dimorphic traits that represent an addition to this existing consensus, firstly reviewing findings reported in other studies and contrasting or not with the observations from our sample. For example, while Celebi et al. [8] reported thinner vermilions in the Italian group, this partially contrasts with the increased protrusion and fullness of the lower vermilion observed in our sample, a discrepancy likely ascribable to diverse methodological approaches. Another trait reported by several authors across different ethnicities (i.e., Egyptians or European descendants) is the deeper labial philtrum in males [8,11]. This finding contrasts with the overall protrusion of the upper lip observed in the present study and others [14,33,41], which aligns with the skeletal evidence that males tend to be more prognathic than females [36]. Finally, the inconsistencies regarding the wider mandibles in males [6,11,41] and the upturned nasal tip in females [6,11,20,41], neither of which were observed in our sample, may reflect variations in ethnicity, age range, or specific methodologies across studies. When considering pure morphological shape differences, the males in our cohort did not display wider mandibles. This trait may be masked by the greater cheek/malar fullness observed in females, which extends from the infraorbital region to the buccal and mandibular areas. This accentuated fullness may be influenced by confounding factors such as a higher Body Mass Index (BMI) and increased facial adipose tissue. As data regarding these specific variables were unavailable, we were unable to control their potential effects on the observed morphology. Furthermore, while several authors have identified an upturned nasal tip as a female characteristic in other populations [6,11,41], this trait was not detected in our Italian sample. Such inconsistency likely underscores the influence of diverse ethnicity on sexual dimorphic expression.

Notably, two sexually dimorphic traits identified in our study have not been reported in previous investigations: wider temples in females and a flatter labiomandibular crease in males. The increased temporal width in females may reflect the underlying frontal bone morphology. Indeed, previous research has noted that “the lateral sides posteriorly behind the linea temporalis were more prominent in females” [44]. The flatter labiomandibular crease in males, similarly to the flatter nasolabial fold identified here and also by Matthews et al. [11], likely results from a combination of soft and hard craniofacial tissue characteristics. We speculate that the flatter appearance of the cheeks in males due to a lower amount of adipose tissue and wider frontal and zygomatic processes [18], coupled with the protrusion of the upper lip and chin caused by skeletal maxillary prognathism [36] and more robust mandible [45,46,47], result in a smoother transition from the cheeks to perioral and mental regions in this sex. Conversely, the opposite pattern is observed in females, where a higher concentration of adipose tissue in the malar regions [43,48] creates more defined facial contours. However, the observed discrepancies should not be interpreted as purely sex-related traits, as their expression could be influenced by factors such as body composition and BMI, variables not verified in the present study. The absence of this data requires caution in interpreting our results concerning sex-related traits.

Although the pattern of sexual dimorphism identified here largely mirrors that of other Caucasian and European-descendent populations, the expression of these localized differences reinforces the need for group-specific studies, especially when developing reference normative models for clinical, forensic, or anthropological purposes. The group-specific characterization of sexually dimorphic traits and the establishment of sex-specific average faces have direct clinical implications for facial gender-affirming surgeries [6]. These procedures aim to align the facial morphology of the patient with their gender (masculinization/feminization) [49], and the data presented in this study are useful as a reference to design personalized surgical plans and evaluate outcomes [6]. In forensic contexts, these data hold important value for age progression/regression techniques, essential for estimating the appearance of long-term missing persons or reconstructing their past appearance [50]. However, despite the robust methodologies employed, it should be acknowledged that our sample included only participants imaged with a neutral expression and without facial hair, which, while a common standard in morphometric analysis, may limit the generalizability of our findings to real-world forensic and clinical contexts that typically lack standardized images. Future investigations may attempt to develop methodologies capable of ‘correcting’ non-ideal images, such as those portraying subjects with facial hair, as proposed by Dhahri et al. [51], or non-neutral expressions, as Matthews et al. [31] did to correct mouth openness in 1-year-old children.

Facial soft tissue sexual dimorphism likely reflects, at least partially, the underlying skeletal morphology. The dimorphic features observed in the upper facial third, particularly at the glabellar and supraorbital ridges, closely mirror established anthropological patterns of the frontal bone [52,53], considered one of the most sexually dimorphic cranial elements [44]. In males, the anterior projection of the lower forehead and larger frontal sinuses [54,55] result in a more sloped profile and a decreased glabellar angle [56]. Similarly, the robust, squarer male chin compared to the tapered female one reflects the sexually dimorphic features of the mandible, whose robustness [45,46,47], when combined with increased maxillary prognathism [36], contributes to the perioral protrusion observable in facial soft tissues. Conversely, the fuller, rounder appearance of the female face is mainly driven by a greater subcutaneous fat volume in the malar region [43,48] rather than skeletal differences. Similarly, anthropological studies evaluating sexual dimorphism of the nose consistently reported males exhibiting bigger noses to meet higher physiological oxygen demands [38,57], rather than differences in the underlying bones even though greater linear dimensions of the pyriform aperture in males have been reported across diverse populations/groups [45]. While our findings align with previous reports on sexual dimorphism in both facial soft tissues and its correlation with the underlying skeleton, a major limitation of the present study is that hard and soft tissues were not analyzed within the same subjects. Therefore, it was not possible to directly evaluate the degree of morphological integration between the craniofacial skeleton and the overlying soft tissues. Future studies should aim to combine skeletal and soft tissue 3D images of the same subjects to clarify the relationship between the two components, ideally by using spatially dense representations of both tissues and multivariate analyses. Additionally, facial morphology undergoes modifications during ontogenetic growth [34,58] and aging [13,43]. However, the onset, duration and rate of changes due to these biological phenomena vary considerably among subjects [33,59], and different facial regions may exhibit distinct developmental/aging trajectories, creating a heterogenous pattern of morphological changes rather than a uniform one [60]. Therefore, longitudinal studies are required to elucidate the timing and progression of facial sexual dimorphism across different life stages. Ideally, such studies should take into account information about factors potentially influencing sex-related traits, such as body composition and BMI, hormonal levels, and genetics, to disentangle their contribution. Addressing these research gaps can be crucial for a comprehensive understanding of facial sexual dimorphism, providing significant clinical, diagnostic, anthropological and forensic implications. Finally, given the shared patterns of sexually dimorphic traits observed between our Italian sample and other European populations, future research should address the comparison of multiple populations and groups using standardized GMM pipelines. Such an approach would enable cross-population comparisons and clarify whether consistent patterns of sexual dimorphism emerge from distinct male and female “archetypal” configurations.

A primary strength of our study is the simultaneous analysis of shape and form, which allowed us to characterize pure morphological shape differences and to evaluate the specific contribution of size to facial sexual dimorphism. Similarly, the combined application of both PCA and PLSR allowed us to evaluate complementary aspects of facial shape and form variation. This dual methodological perspective provides a more robust characterization of facial sex differences, enhancing the applicability of our findings.

## 5. Conclusions

This study presents the first characterization of facial sexual dimorphism in healthy Italian adults using a spatially dense GMM approach applied to analyze both shape and form on 3D stereophotogrammetric images. Our results proved that sex-related differences are evident in both shape and form, and the findings are largely consistent with those already reported in the literature. Nonetheless, we identified specific sexually dimorphic traits unique to this cohort: flatter labiomandibular folds in males, and overall protrusion of the cheeks and the temporal region in females.

To establish population- and age-specific normative data concerning morphological sex differences in the face is useful for diverse applications. In esthetic and maxillofacial surgeries or orthodontics, these data serve as reference to guide the diagnosis of dysmorphisms and plan appropriate interventions. In forensic science, these findings can enhance the accuracy of craniofacial reconstruction and assist in predicting the appearance of missing individuals or in profiling persons from CCTV cameras.

## Figures and Tables

**Table 1:** Results of the PCA for the analysis of shape.

PC	Variance (%)	Cumulative Variance (%)	Eigenvalue
1	24.74	24.74	13,988.46
2	19.82	44.56	11,206.1
3	9.39	53.95	5306.89
4	7.66	61.61	4329.61
5	4.79	66.40	2709.04
6	2.81	69.21	1586.44
7	2.48	71.69	1404.15
8	2.36	74.05	1336.85
9	2.16	76.21	1218.74
10	1.87	78.08	1055.57
11	1.64	79.72	927.81
12	1.38	81.10	780.48
13	1.26	82.36	714.43
14	1.17	83.53	663.93
15	1.14	84.67	647.05

**Table 2:** Results of the PCA for the analysis of form.

PC	Variance (%)	Cumulative Variance (%)	Eigenvalue
1	45.28	45.28	50,313.61
2	14.06	59.34	15,620.72
3	10.58	69.92	11,757.93
4	4.97	74.89	5517.21
5	3.71	78.60	4121.79
6	2.58	81.18	2861.61
7	1.54	82.72	1716.08
8	1.39	84.11	1545.34
9	1.30	85.41	1445.06

**Table 3:** Summary of the findings about facial sexual dimorphism across studies using 3D spatially dense geometric morphometrics.

First Author, Year	Population/Group	Methodology, Statistics	Study-Specific Findings	Common Findings
Hennessy et al., 2005 [41]	Irish, Scottish, Welsh, English	Shape, Hotelling’s T^2^, color maps	F: upturned nasal tip	M: larger faces (length and width), supraorbital bossing, deep-set eyes, bigger dimensions of the nose, protrusion of the upper lip and chin, and wider mandible F: smaller and rounder face with malar fullness and vertical profile of the forehead M: larger faces (length and width), supraorbital bossing, deep-set eyes, bigger dimensions of the nose, protrusion of the upper lip and chin, and wider mandible F: smaller and rounder face with malar fullness and vertical profile of the forehead
Claes et al., 2014 [25]	European descendants (USA, Brazil), West African (Cape Verde)	Shape, BRIM, color maps	R^2^ sex effect on shape: 13%	
Velemínská et al., 2022 [33]	Czech	Form, Linear distances, two-way ANOVA, GMM, per-vertex two-sample *t*-tests, color maps	M: protrusion of the labial philtrum	
Bannister et al., 2022 [6]	European descendants	Shape and Form, Linear distances, Welch’s *t*-test, GMM, PLSR, color maps	R^2^ sex effect on shape: 6% R^2^ sex effect on form: 30% M: face size 7.3% bigger, longer upper lip F: upturned nasal tip and narrower nostrils	
Matthews et al., 2023 [11]	European descendants	Shape, GMM, PLSR, color maps	M: deeper labial philtrum F: deeper nasolabial folds, upturned nasal tip	
Da Silva et al., 2025 [14]	European descendants	Shape, GMM, sex-on-shape regression, color maps	R^2^ sex effect on shape: 3.5% R^2^ sex effect on shape (non-allometric component): 1.7%	
Our study	Italian	Shape and Form, GMM, PLSR, color maps	R^2^ sex effect on shape: 10.4% R^2^ sex effect on form: 24.2% M: face size 5.3% bigger, flatter labiomandibular crease, absence of wider mandible F: wider temples, fuller cheeks extending from the infraorbital region to the buccal and mandibular ones	

M: male; F: female; 3D; three-dimensional; GMM: geometric morphometric; PLSR: Partial Least Squares Regression; BRIM: Bootstrapped Response-based Imputation Modeling.

## Data Availability

The data that support the findings of this study are not publicly available due to privacy or ethical restrictions.

## Supplementary Materials

The following supporting information can be downloaded at: https://www.mdpi.com/article/10.3390/diagnostics16050712/s1, Table S1: Detailed summary of the findings about facial sexual dimorphism across studies.

## Abbreviations

The following abbreviations are used in this manuscript:

2D	Two-dimensional
3D	Three-dimensional
CCTV	Closed-circuit television
CS	Centroid size
GMM	Geometric morphometric
GPA	Generalized Procrustes analysis
PC	Principal component
PCA	Principal component analysis
PLSR	Partial least squares regression
