# Supplementary material for: How Sex Shapes Facial Morphology in Adults: A 3D Geometric Morphometric Study
Source: Diagnostics (Basel). 2026 Feb 27;16(5):712. doi: 10.3390/diagnostics16050712 (PMC12984368; doi:10.3390/diagnostics16050712)
Supplement: Supplementary file 1 [file diagnostics-16-00712-s001.zip › diagnostics-4130698-supplementary.pdf]

Table S1 – Detailed summary of the findings about facial sexual dimorphism across studies

<table>
<tr><td colspan="9">Studies using sparse-landmark approach</td></tr>
<tr><td rowspan="2">First author, Year</td><td rowspan="2">Population/Group</td><td rowspan="2">Sample size (F, M)</td><td rowspan="2">Age range (mean)</td><td colspan="3">Analysis</td><td colspan="2">Results</td></tr>
<tr><td colspan="2">Material</td><td>Methodology, Statistics</td><td>Study-specific findings</td><td>Common findings</td></tr>
<tr><td>Ferrario et al.,
1993 [20]</td><td>White Caucasian</td><td>108
(51F, 57M)</td><td>20-27 yrs</td><td colspan="2">2D photographs</td><td>Euclidean Distance Matrix Analysis (EDMA),
T-statistics</td><td>Sex dimorphism due to the different size and spatial position of similarly shaped facial structures

M: rectangular face
F: squarer face, upturned nasal tip</td><td rowspan="3">M: larger face (length and width), prominent and wider noses, wider mouths

F: smaller face</td></tr>
<tr><td>Ferrario et al.,
1994 [21]</td><td>White Caucasian</td><td>76
(36F, 40M)</td><td>19-32 yrs
(21 yrs)</td><td colspan="2">3D coordinates</td><td>Euclidean Distance Matrix Analysis (EDMA),
T-statistics</td><td>M: face size 6% to 7% bigger</td></tr>
<tr><td>Celebi et al.,
2018 [8]</td><td>Italian

Egyptian</td><td>139
(67F, 72M)

120
(60F, 60M)</td><td>18-30 yrs</td><td colspan="2">3D images</td><td>Linear measurements, independent t-tests</td><td>Italians M: thinner lip vermilions height

Egyptians M: deeper labial philtrum and bigger lip dimensions</td></tr>
<tr><td colspan="9">Studies using sparse-landmark and spatially dense GMM approaches in 3D</td></tr>
<tr><td rowspan="2">First author, Year</td><td rowspan="2">Population/Group</td><td rowspan="2">Sample size (F, M)</td><td rowspan="2">Age range (mean)</td><td colspan="3">Analysis</td><td colspan="2">Results</td></tr>
<tr><td colspan="2">Material</td><td>Methodology, Statistics</td><td>Study-specific findings</td><td>Common findings</td></tr>
<tr><td>Velemínská et al.,
2022 [33]</td><td>Czech</td><td>456
(250F, 206M)</td><td>14-83 yrs</td><td>3D images</td><td>Shape</td><td>Linear distances, two-way ANOVA</td><td>M: protrusion of the labial philtrum</td><td>M: larger faces (length and width), supraorbital bossing, deep-set eyes, bigger</td></tr>
</table>

						GMMs, per-vertex two-sample t-tests, Color maps		dimensions of the nose, protrusion of the upper lip and chin, and wider mandible<br><br>F: smaller and rounder face with malar fullness and vertical profile of the forehead
Bannister et al., 2022 [6]	European descendants	1573 (1028F, 545M)	> 20 yrs	3D images	Shape<br><br>Form	Linear distances, Welch's t-test<br><br>GMMs, PLSR, color maps	R^2 sex effect on shape: 6%<br>R^2 sex effect on form: 30%<br><br>M: face size 7.3% bigger, longer upper lip<br>F: upturned nasal tip and narrower nostrils	
Studies using spatially dense GMM approaches in 3D								
First author, Year	Population/Group	Sample size (F, M)	Age range (mean)	Analysis			Results	
				Material		Methodology, Statistics	Study-specific findings	Common findings
Hennessy et al., 2005 [41]	Irish, Scottish, Welsh, English	128 (82F, 46M)	F: 20-59 yrs (32.2 ± 9.2 yrs)<br>M: 22-65 yrs (33.1 ± 9.7 yrs)	3D images	Shape	Hotelling's T^2, color maps	F: upturned nasal tip	M: larger faces (length and width), supraorbital bossing, deep-set eyes, bigger dimensions of the nose, protrusion of the upper lip and chin, and wider mandible<br><br>F: smaller and rounder face with malar fullness and vertical profile of the forehead
Claes et al., 2014 [25]	European descendants (USA, Brazil), West African (Cape Verde)	592	18-40 yrs	3D images	Shape	BRIM, color maps	R^2 sex effect on shape: 13%	
Mydlová et al., 2015 [18]	Czech	194 (115F, 79M)	20-82 yrs	3D images	Shape<br><br>Form	Hotelling's T^2, color maps	/	
Matthews et al., 2023 [11]	European descendants	2446 (1223F, 1223M)	18-84 yrs (27.8 yrs)	3D images	Shape	GMMs, PLSR, color maps	M: deeper labial philtrum	

							F: deeper nasolabial folds, upturned nasal tip	
Da Silva et al.,<br>2025 [14]	European descendants	1080<br>(540F, 540M)	18-40 yrs<br>(26.1 ± 5.3 yrs)	3D images	Shape	GMMs,<br>sex-on-shape regression, color maps	R^2 sex effect on shape: 3.5%<br>R^2 sex effect on shape (non-allometric component): 1.7%	
Our study	Italian	342<br>(170 F, 172 M)	F: 18-40 yrs<br>(25.7 ± 7.2 yrs)<br>M: 18-40 yrs<br>(23.2 ± 5.8 yrs)	3D images	Shape<br><br>Form	GMMs, PLSR, color maps	R^2 sex effect on shape: 10.4%<br>R^2 sex effect on form: 24.2%<br><br>M: face size 5.3% bigger, flatter labiomandibular crease, absence of wider mandible<br>F: wider temples, fuller cheeks extending from the infraorbital region to the buccal and mandibular ones	

M: male; F: female; 2D: two-dimensional; 3D; three-dimensional; GMM: geometric morphometric, PLSR: partial least square regression, BRIM: bootstrapped response-based imputation modeling.
